# Gene conversion events and variable degree of homogenization of rDNA loci in cultivars of *Brassica napus*

**DOI:** 10.1093/aob/mcw187

**Published:** 2016-10-05

**Authors:** Jana Sochorová, Olivier Coriton, Alena Kuderová, Jana Lunerová, Anne-Marie Chèvre, Aleš Kovařík

**Affiliations:** 1 Laboratory of Molecular Epigenetics, Institute of Biophysics, Královopolská 135, 61265 Brno, Czech Academy of Science, v.v.i., Czech Republic; 2 Institut National de la Recherche Agronomique (INRA), UMR 1349 IGEPP, BP 35327, F-35653 Le Rheu cedex, France

**Keywords:** *Brassica napus*, allopolyploidy, rDNA, chromosome evolution, gene conversion

## Abstract

**Background and aims** *Brassica napus* (AACC, 2*n* = 38, oilseed rape) is a relatively recent allotetraploid species derived from the putative progenitor diploid species *Brassica rapa* (AA, 2*n* = 20) and *Brassica oleracea* (CC, 2*n* = 18). To determine the influence of intensive breeding conditions on the evolution of its genome, we analysed structure and copy number of rDNA in 21 cultivars of *B. napus*, representative of genetic diversity.

**Methods** We used next-generation sequencing genomic approaches, Southern blot hybridization, expression analysis and fluorescence *in situ* hybridization (FISH). Subgenome-specific sequences derived from rDNA intergenic spacers (IGS) were used as probes for identification of loci composition on chromosomes.

**Key Results** Most *B. napus* cultivars (18/21, 86 %) had more A-genome than C-genome rDNA copies. Three cultivars analysed by FISH (‘Darmor’, ‘Yudal’ and ‘Asparagus kale’) harboured the same number (12 per diploid set) of loci. In *B. napus* ‘Darmor’, the A-genome-specific rDNA probe hybridized to all 12 rDNA loci (eight on the A-genome and four on the C-genome) while the C-genome-specific probe showed weak signals on the C-genome loci only. Deep sequencing revealed high homogeneity of arrays suggesting that the C-genome genes were largely overwritten by the A-genome variants in *B. napus* ‘Darmor’. In contrast, *B. napus* ‘Yudal’ showed a lack of gene conversion evidenced by additive inheritance of progenitor rDNA variants and highly localized hybridization signals of subgenome-specific probes on chromosomes. *Brassica napus* ‘Asparagus kale’ showed an intermediate pattern to ‘Darmor’ and ‘Yudal’. At the expression level, most cultivars (95 %) exhibited stable A-genome nucleolar dominance while one cultivar (‘Norin 9’) showed co-dominance.

**Conclusions** The *B. napus* cultivars differ in the degree and direction of rDNA homogenization. The prevalent direction of gene conversion (towards the A-genome) correlates with the direction of expression dominance indicating that gene activity may be needed for interlocus gene conversion.

## INTRODUCTION

*Brassica napus* (AACC, 2*n* = 38) has been intensively cultivated since the middle of the 20th century (Baranyk and Fábry, 1999), and is a natural post-Neolithic allotetraploid species that formed approx. 7500 years ago (Chalhoub *et al.*, 2014). Numerous cytogenetic, genetic and genomic studies showed that *B. napus* formed from crosses between *B. oleracea* (CC, 2*n* = 18) and *B. rapa* (AA, 2*n* = 20). Its polyphyletic origin was established from chloroplast haplotypes suggesting that *B. napus* could have been formed at least twice from crosses between different forms of progenitor species (Palmer *et al.*, 1983; Erickson *et al.*, 1983; Allender and King, 2010). Furthermore, cytogenetic studies using genomic *in situ* hybridization (GISH) (Howell *et al.*, 2008) or GISH-like BAC probes (Leflon *et al.*, 2006) have confirmed that the A and C genomes have largely remained distinct in *B. napus* and showed disomic inheritance of its chromosomes. However, comparative genetic mapping studies have demonstrated that homeologous recombination occurred between the A and C genomes generating different translocations between the genomes (Parkin *et al.*, 1995; Osborn *et al.*, 2003; Piquemal *et al.*, 2005; Udall *et al.*, 2005; Alix *et al.*, 2008; Chalhoub *et al.*, 2014) indicating that disomic inheritance may be occasionally compromised perhaps as a result of increased transposon element activity in the newly formed allopolyploid nucleus (Sarilar *et al.*, 2013; and reviewed by Tayalé and Parisod, 2013).

The rDNA locus encoding ribosomal 18S, 5·8S and 26S rRNA genes (35S rDNA) has been used in numerous cytogenetic and phylogenetic studies (Poczai and Hyvonen, 2010). It is somewhat paradoxical that, despite conservativity of genes (coding regions), it is one of the most dynamic objects in the genome. This is manifested by sequence variation of intergenic (IGS) (Borisjuk *et al.*, 1997) and internal transcribed (ITS) spacers (Alvarez and Wendel, 2003; Nieto Feliner and Rosselló, 2007), and frequent changes in number and position of loci on chromosomes (Dubcovsky and Dvorak, 1995). In many hybrids and allopolyploids only one parental copy is found while the other is often reduced or lost (Volkov *et al.*, 1999; Kovarik *et al.*, 2005; Doyle *et al.*, 2008; Weiss-Schneeweiss *et al.*, 2012; Mahelka *et al.*, 2013; and reviewed by Volkov *et al.*, 2007; Buggs *et al.*, 2012; Weiss-Schneeweiss *et al.*, 2013). Homogenization may be mediated by different genetic events including locus loss, reduction of copies or replacement with newly amplified ones (Nieto Feliner and Rosselló, 2012). Theoretical models suggest that non-homologous recombination and gene conversion are the mechanisms driving rDNA homogenization processes (Zimmer *et al.*, 1980; Dover, 1982).

The genus *Brassica* is known to harbour large variability in the number of rDNA loci, ranging between two and five per haploid set (Maluszynska and Heslop-Harrison, 1993; Hasterok *et al.*, 2006; PlantrDNAdatabase – http://www.plantrdnadatabase.com/, Garcia *et al.*, 2012). *Brassica rapa*, the presumed progenitor species of *B. napus*, has five loci (two major and three minor) per haploid set while the second presumed genome donor, *B. oleracea*, harbours two loci (Maluszynska and Heslop-Harrison, 1993). At the cytogenetic level, the cultivars of *B. napus* show genotypic differences in number, distribution and morphology of rDNA chromosomal loci (Snowdon *et al.*, 1997; Fukui *et al.*, 1998; Hasterok *et al.*, 2001, 2006; Ali *et al.*, 2005; Xiong and Pires, 2011; Amosova *et al.*, 2014). Previous Southern blot hybridization revealed restriction fragments corresponding to both parents indicating Mendelian inheritance of rDNA in *B. napus* (Bennett and Smith, 1991; Waters and Schaal, 1996). However, several cytogenetic observations suggest that rDNA structural changes occurred in *B. napus*. First, both natural and synthetic lines show a reduced number of loci compared to the sum of progenitor loci (Maluszynska and Heslop-Harrison, 1993; Snowdon *et al.*, 1997; Kulak *et al.*, 2002; Hasterok *et al.*, 2006). Variability in the number of rDNA loci seems to be limited to minor loci (mostly in the A genome) while major nucleolus organizer regions (NORs) on chromosomes A1, A3, C7 and C8 seem to be intact – for ribosomal RNA genes. We followed the nomenclature allowing attribution of each chromosome to a linkage group in *B. rapa* (Kim *et al.*, 2009) and *B. oleracea* (Howell *et al.*, 2002) (www.brassica.info). Second, the rDNA fluorescence *in situ* hybridization (FISH) signals to C-genome NORs were weaker on the C-genome NORs compared to those in parental *B. oleracea* (Xiong and Pires, 2011). Third, the C-genome rDNA sites were not totally blocked by the *B. oleracea*-specific IGS probe (Howell *et al.*, 2008). Finally, chromosome banding showed interpopulation variation in the amount and distribution of heterochromatin adjacent to or overlapping with NORs (Amosova *et al.*, 2014).

In the present study, to shed more light on the evolutionary patterns of rDNA, we carried out a population-level study of gene copies and loci in 21 cultivars of *B. napus*. We posed the following questions: (1) Are homeologous rRNA genes and loci faithfully inherited in all populations of the allotetraploid? (3) Is the expression status of homeologues additive or biased in different organs according to the structure of the variety? To address these questions, we combined structural and functional analyses. We obtained evidence for gene conversion of thousands of C-genome units that are being replaced by the A-genome type units in cultivar ‘Darmor’. This does not seem to occur in another cultivar ‘Yudal’ where loci and genes remain intact. Variation between cultivars suggests a tentative establishment of bidirectional homogenization amongst the post-Neolithic *B. napus* allotetraploid.

## MATERIALS AND METHODS

### Plant material

Oilseed rape cultivars were chosen according to the genetic diversity of the species with mainly ssp. *oleifera* cultivars but also two accessions of ssp. *rapifera* (‘Rutabagas 22’ and ‘Rutabaga 95’) and one of ssp. *pabularia* (‘Asparagus kale’). Fourteen spring *B. napus* cultivars (‘Asparagus kale’, ‘Brutor’, ‘Loras’, ‘Nachan’, ‘Norin 1’, ‘Norin 6’, ‘Norin 9’, ‘Norin 10’, ‘Oro’, ‘Spok’, ‘Stellar’, ‘Taichung’, ‘Yudal’ and ‘Westar’) and seven winter cultivars (‘Darmor’, ‘Maxol’, ‘Mohican’, ‘Petranova’, ‘Rutabaga 22’, ‘Rutabaga 95’ and ‘Tapidor’) were used for genetic and expression analysis of rDNA (Supplementary Data Table S1). As controls of presumed diploid progenitors, we used Z1, a doubled haploid line of *B. rapa* provided by AAFC, Canada, and HDEM, a doubled haploid line of *B. oleracea* provided by BrACySol BRC, Ploudaniel, France. Plants were grown from seeds in a greenhouse. Most seeds were obtained from INRA BrACySol BRC, Ploudaniel, France. Seeds of *B. napus* ‘Tapidor’ were a gift from the laboratory of Functional Genomics and Proteomics of Plants, CEITEC, Brno, Czech Republic, and were originally obtained from Ian Bancroft’s laboratory, the John Innes Centre (JIC), Norwich, UK.

### Southern blot hybridization

Southern blotting followed the protocol described by Koukalova *et al.* (2010) using rDNA probes, A-genome-specific IGS probe (IGS-A) and C-genome-specific IGS probe (IGS-C) labelled with ^32^P (DekaPrime kit, Fermentas, Lithuania). The hybridization signals were visualized by Phosphor imaging (Typhoon 9410, GE Healthcare, PA, USA) and signals were quantified using ImageQuant software (GE Healthcare).

### Fluorescence *in situ* hybridization

Preparation of slides and hybridization were carried out according to procedures detailed by Suay *et al.* (2014). The ribosomal probe used in this study was 35S rDNA (pTa 71 clone) from wheat (Gerlach and Bedbrook, 1979), IGS-A and IGS-C probes described further below and the BAC clone *B. oleracea* named Bob014O06 (Howell *et al.*, 2002). This BAC clone was used as ‘GISH-like’ to distinguish specifically all C-genome chromosomes in *B. napus* (Suay *et al.*, 2014). The 35S rDNA and BAC clone were labelled with Alexa-488 dUTP by random priming, the IGS-A with biotin-dUTP (Roche, Mannheim, Germany) using PCR and the IGS-C with biotin-dUTP (Roche) using nick translation (Bionick DNA labelling System, Thermo Fisher Scientific, Waltham, MA, USA). Biotinylated probe was immunodetected by Texas Red avidin DCS (Vector Laboratories, Burlingame, CA, USA) and the signal was amplified with biotinylated anti-avidin D (Vector Laboratories). The chromosomes were mounted and counterstained in Vectashield (Vector Laboratories, Ontario, Canada) containing 2·5 μg mL^–1^ 4′,6-diamidino-2-phenylindole (DAPI). Fluorescence images were captured using a CoolSnap HQ camera (Photometrics, Tucson, AZ, USA) on an Axioplan 2 microscope (Zeiss, Oberkochen, Germany) and analysed using MetaVue^TM^ (Universal Imaging Corporation, Downington, PA, USA).

### IGS amplification and subgenome-specific probe generation

Genomic DNA was extracted by a CTAB method from fresh leaves (Saghai-Maroof *et al.*, 1984). IGS regions were amplified by PCR using primers Pr1 (AGACGACTTTAAATACGCGAC) (Garcia and Kovarik, 2013) and a newly designed reverse BrasProm_R primer annealing to promoter (GAGTGCCTACCCCTTATA). Amplification was performed using the following programme: 92 °C for 20s, 62·4 °C for 30 s, 70 °C for 3 min, all for 35 cycles followed by 8 min of extension at 70 °C. The PCR products were separated on an agarose gel, cut and ligated to a pDrive plasmid (Qiagen, Germany). Recombinant clones were amplified and subcloned after restriction. The following restriction enzymes were used: *Msc*I and *Eco*RI (long IGS variant from ‘Darmor’), *Msc*I and *Eco*RI (short IGS variant from ‘Darmor’), *Hinc*II (‘Asparagus Kale’), *Msc*I and *Acc*I (‘Yudal’). The fragments were ligated to a pDrive vector and sequenced. Sequences were submitted to GenBank under accession numbers KT008109 (‘Darmor’ L), KT008110 (‘Darmor’ S), KT008111 (‘Asparagus kale’) and KT008112 (‘Yudal’). Subrepeats were analysed using the YASS genomic similarity search tool (Noe and Kucherov, 2005) and Tandem Repeats Finder (Benson, 1999). A phylogenetic tree was reconstructed using the Seaview program (Gouy *et al.*, 2010).

The A-genome-specific probe (IGS-A) was prepared by digestion of a plasmid DNA of a *B. rapa* IGS clone (GenBank: KT008109) with *Tru*1I. The resulting 1·4-kb *Tru*1I fragment was cloned and sequenced (pBrapsr2 clone). Its 1436-insert was found to contain the majority of the *B. rapa*-specific C-subrepeats and part of the B-subrepeats. We checked the specificity by mapping the next-generation sequencing (NGS) reads to the IGS-A sequence. About 1·2 % *B. rapa* Illumina reads (see further below) were mapped to the insert. There were no longer (>60 bp) regions covered by NGS reads from *B. oleracea*, and the region between nucleotide positions 400 and 1300 showed essentially no hits. The C-genome-specific probe (IGS-C) was prepared by PCR amplification using newly designed IGS primers Oler_F1 (TGACGGACAGTCCTCGTG) and Oler_F2 (CAGTACACATATCAGCACG). The primers were derived from a *B. oleracea* IGS repetitive subregion 400–1300 positions downstream from the 26S gene to which essentially only *B. oleracea* and not *B. rapa* NGS reads were mapped. In a PCR, *B. oleracea* DNA template was used at a low concentration (∼1 ng per reaction). The resulting product was purified and labelled without further subcloning.

### Expression analysis

The procedures followed those described by Ksiazczyk *et al.* (2011). Briefly, total RNA was isolated using the RNeasy Plant Mini Kit (Qiagen, Hilden, Germany) and contaminating DNA was removed by TURBO™ DNase (Ambion, Austin, TX, USA). Reverse transcription reaction was performed in 40 μL volume and contained: 2 μg RNA, 4 pmol random primers (N_9_) and 200 U reverse transcriptase (Invitrogen Superscript II RNase H, Paisley, UK), according to conditions recommended by the supplier. Expression of homeologous genes was carried out using a cleaved amplified polymorphism sites (CAPS) assay. The ITS1 region was amplified using 0·5 μL cDNA and primers 18Sfor and 5·8Srev (Kovarik *et al.*, 2005). PCR products were digested with restriction enzyme *Rsa*I in an amplification mixture, separated by electrophoresis and visualized by ethidium bromide staining.

### *In silico* rDNA sequence assembly

Regions (18S, ITS, 26S) of 35S rDNA of *B. napus*, *B. oleracea* and *B. rapa* were assembled into contigs by mapping of Illumina reads to reference 18S–ITS1–5·8S–ITS2–26S sequences obtained from GenBank (AF128100.1, KM538957, KF704394, GQ891874, X51576.1, X13557.1). The 35S rDNA sequence of *Arabidopsis thaliana* (accession number: X52322) was used as a reference to aid assembly. The following Illumina reads archives were used: *B. napus* ‘Darmor’ (ERR457740, ERR457754 and ERR457738), *B. napus* ‘Yudal’ (ERR457757), *B. rapa* (SRR1296482 and SRR5479996) and *B. oleracea* (SRR074124). Sequence downloads and basic read manipulations of genomic reads were done with the aid of the Galaxy Server (Goecks *et al.*, 2010). Different regions of 35S rDNA sequences (contigs) were ultimately assembled into one single consensus sequence for each species using CLC Genomics Workbench 6.5.1 (Qiagen, Aarhus, Denmark) via default settings. There were no significant differences in coverage between individual regions and most nucleotides were read >130 times.

### Intragenomic variation of rDNA determined from NGS reads

CLC Genomics Workbench 6.5.1 was used to estimate intragenomic variation among 35S rDNA units in *B. napus*, *B. oleracea* and *B. rapa*. Before analysis of single nucleotide polymorphisms (SNPs) all reads with Ns and all reads less than 90 nt in length were removed. In some cases, the number of reads was sampled below 100 million to decrease computing time. Illumina reads used were first mapped to the 35S rDNA consensus sequence of *B. oleracea* with the following mapping settings: mismatch cost value 2, insertion cost value 3, deletion cost value 3, with both the length fraction value and the similarity fraction value set to 0·8. Variations were then detected via the Probabilistic Variant Detection function tool in CLCbio, using default settings. SNPs were filtered as follows: minimum read coverage 100, Count (the number of countable reads supporting the allele) 10, frequency (the ratio of ‘the number of “countable” reads supporting the allele’ to ‘the number of “countable” reads covering the position of the variant’) ≥20 % (high-frequency SNPs) or 5–20 % (medium-frequency SNPs). Comparative analysis of rDNA variants was carried out using tools with the COMPARE function of CLC Genomics Workbench.

ITS amplicons were obtained by emulsion PCR and sequenced on a Roche 454 GS-FLX platform (EuroFins MWG, Ebersberg, Germany). The reads were mapped to the 256 bp of ITS1 (*B. oleracea* and *B. rapa*) and sorted into clusters according to the procedures described by Matyasek *et al.* (2012). The cut off for scoring SNPs was set to ≥ 10 % reads.

## RESULTS

### Variability of rDNA homeologue ratios in *B. napus* cultivars

**Fig. 1.**
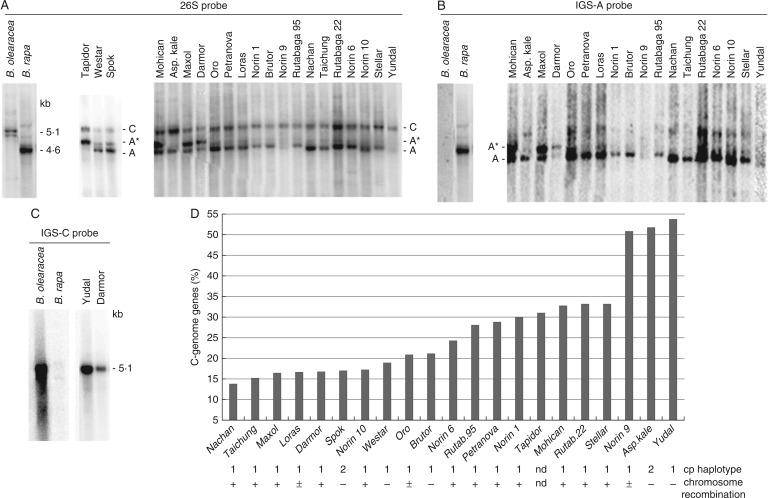
(A) Southern blot hybridization of genomic DNA from several *B. napus* cultivars. In the left panel the blot was hybridized with the 26S probe. After stripping the blot was rehybridized with the A-genome-specific IGS probe (B). ‘C’, C-genome bands; ‘A’, A-genome bands; ‘A*’ indicates an IGS family amplified in a subset of *B. napus* cultivars. (C) Example of a Southern hybridization of the C-genome-specific IGS probe. Note strong hybridization of the probe to ‘Yudal’ DNA. (D) The radioactivity of bands was quantified using a Phosphorimager and homeologue gene number expressed as a proportion of C-genome rDNA to total rDNA. Numbers below indicate two major chloroplast haplotypes identified by Cifuentes *et al.* (2010). In the same study, the *B. napus* cultivars were divided into groups with low (–), intermediate (±) and high (+) frequency of homeologue chromosome pairing in meiosis (symbols towards the bottom).

To determine homeologous genes ratios, we carried out Southern blot hybridization of genomic DNA from 21 cultivars of *B. napus* representing most of the crop diversity, and the presumed diploid progenitor species (Fig. 1). In *B. oleracea* the 26S probe hybridized to a doublet of the *Bst*NI fragments migrating in the upper part of the gel (Fig. 1A). The large size of the *B. oleracea*-specific band is consistent with a longer IGS in this species compared to *B. rapa* (Delseny *et al.*, 1990). In *B. rapa*, there was a single fast-migrating fragment. All *B. napus* cultivars inherited major progenitor fragments: the 4·6-kb ‘A’ inherited from *B. rapa* and the larger 5·1-kb ‘C’ inherited from *B. oleracea*. A minor *B. oleracea* fragment was not detected. Four cultivars (‘Darmor’, ‘Maxol’, ‘Mohican’ and ‘Tapidor’) showed a strong ‘A*’ fragment with the size falling between the ‘A’ and ‘C’ bands. The ‘A*’ fragment hybridized strongly with the A-genome-specific IGS-A probe (Fig. 1B) revealing its A-genome origin. The C-genome-specific IGS-C probe hybridized to the upper 5·1-kb ‘C’ band in *B. oleracea* and *B. napus* while it did not hybridize with *B. rapa* and derived A-genome bands of *B. napus* (Fig. 1C and Supplementary Data Fig. S1). The ratio between the C- and A-genome-specific fragments was determined by quantification of Southern blot bands (Fig. 1A) by counting of radioactivity in ‘A’, ‘A*’ and ‘C’ bands using a Phosphorimager. The proportion of C-genome copies calculated as ‘C’/‘C’ + ‘A’ + ‘A*’ percentages varied from 14 % in *B. napus* ‘Nachan’ to 54 % in ‘Yudal’ (Fig. 1D). The ‘A’+ ‘A*’/‘C’ band ratio ranged from 0·85 to 6·14 with a median of 3·10. The cultivars analysed in this study were previously genotyped by chloroplast markers (Cifuentes *et al.*, 2010). Two major chloroplast haplotypes are indicated by numbers below the graph (Fig. 1D). There was no apparent relationship to rRNA gene ratios. The frequency of homeologue chromosome pairing in meiosis differed between cultivars (Cifuentes *et al.*, 2010). Cultivars with high A/C ratios seem to belong to a group harbouring a high frequency of homeologue pairing (+ labels below the graph).

### Sequence polymorphisms of rDNA units

**Fig. 2.**
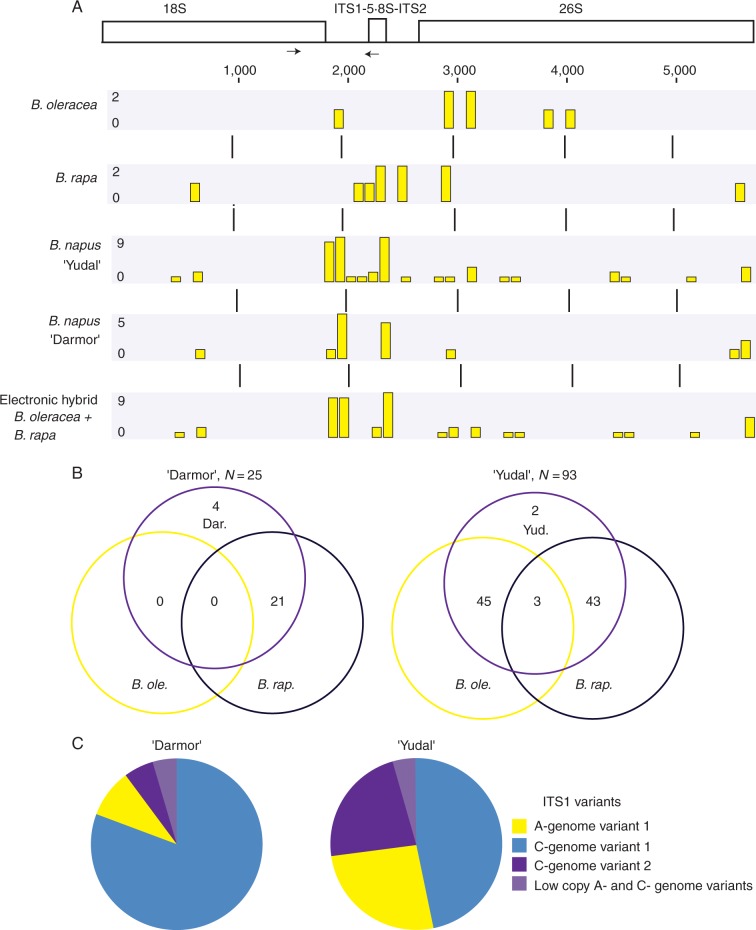
Intragenomic heterogeneity of rDNA units in progenitor species and two *B. napus* cultivars determined from whole genomic Illumina reads (A, B) and Roche 454 sequencing of ITS1 amplicons (C). The graphs in A reflect distribution of highly polymorphic sites (>20 % frequency) along the 18S–5·8S–26S region. Each column represents one or more high-frequency SNPs. Coalescence of more SNPs within the 10-bp window is reflected by column height. (B) Venn diagrams showing comparison of rDNA variants between *B. napus* and its progenitors. Note absence of *B. oleracea* variants in *B. napus* ‘Darmor’. (C) Circle chart diagrams showing the distribution of ITS1 variants in the genomes. The scheme of rDNA unit is depicted in A. Arrows indicate positions of primers used in amplicon sequencing and RT-CAPS analysis.

The availability of genomic reads allowed us to reconstruct 18S–5·8S–26S operons in *B. napus* and the diploid species *B. rapa* and *B. oleracea* (Table 1). The reads were mapped to the reference sequence which had been assembled from sequences in GenBank (for accession numbers, see Methods). All three rRNA genes (18S, 5·8S and 26S) were recovered including intervening ITS1 and ITS2 sequences. From the mapped reads, we generated a single rDNA consensus sequence for each diploid species and two cultivars of *B. napus* (‘Darmor’ and ‘Yudal’). The total length of the recovered 18S–5·8S–26S region was 5803 bp in all samples. Pairwise alignment of consensus sequences revealed 72 polymorphic sites (1·24 % divergence) between *B. rapa* and *B. oleracea* units. *Brassica rapa* had a slightly higher intragenomic variation [ten high- (≥20 %) frequency SNPs per unit] than *B. oleracea* (seven high-frequency SNPs per unit) which may be related to several minor rDNA loci in the *B. rapa* genome. Although most variation occurred in the ITS regions, several significant polymorphisms were also found in the 18S and 26S coding regions (Fig. 2A). The consensus sequences constructed from NGS reads of two *B. rapa* cultivars [sequence archives SRR1296482 and SRR5479996 (susbsp. *pekinensis*)] were 100 % identical while there were differences in quantitative representation of individual nucleotides at eight polymorphic sites (not shown).

**Table 1:** Copy number of rRNA genes in *B. napus* and progenitor species determined from NGS reads

	Read archive accession	Mapped reads*	Total reads	GP^†^ (%)	rDNA (Mb)	Copies^‡^ 1C
*B. rapa*	SRR1296482	1 453 334	78 528 998	1·86	5·80	1709
*B. oleracea*	SRR074124	1 437 383	146 402 687	1·06	5·78	1162
*B. napus* ‘Darmor’	ERR457740, ERR457754, ERR457738	838 265	60 083 363	1·39	11·68	2832
*B. napus* ‘Yudal’	ERR457757	780 687	72 174 366	1·08	9·08	2201

* All consensus sequences were 5803 bp long and included the complete 18S–ITS1–5·8S–ITS2–26S subregion.

† Genome proportion (GP) is defined as number of reads mapped to rDNA divided by the number of total reads (as a percentage). It would be ∼60 % larger if IGS regions are considered.

‡ From genome proportions copy numbers of rDNA sequences were estimated according to the formula: genome size × genome proportion of rDNA units. The following genome sizes were considered: *B. napus*, 1182 Mb; *B. oleracea*, 630 Mb; *B. rapa*, 530 Mb (Bennett and Leitch, 2012).

The distribution of high-frequency SNPs in *B. napus* cultivars is depicted in Fig. 2A. The SNP profiles differed dramatically between ‘Darmor’ and ‘Yudal’ cultivars: *B. napus* ‘Yudal’ had a complex mutation profile similar to that of the virtual hybrid [constructed from 1 : 1 mixing of rDNA reads from the presumed *B. oleracea* (SRR074124) and *B. rapa* (SRR1296482) progenitors]. In contrast, *B. napus* ‘Darmor’ had fewer SNPs and a relatively smooth mutation profile resembling that of *B. rapa*. The high-frequency variants were at least three-fold more abundant in *B. napus* ‘Yudal’ than in *B. napus* ‘Darmor’ (Fig. 2B). The variants from the presumed progenitors were inherited at comparable ratios in *B. napus* ‘Yudal’. In contrast, *B. napus* ‘Darmor’ had 80 % variants inherited from *B. rapa*, 0 % from *B. oleracea* and 20 % were unique. Nevertheless, the C-genome variants were found at low copy (<20 % frequency) in *B. napus* ‘Darmor’ (see further below). Similar results were obtained when analysing promoter regions. For example, the *B. oleracea* promoter had a C nucleotide at position −7 while *B. rapa* had A at this position. In ‘Yudal’, 58 % of reads had A (haplotype inherited from *B. rapa*) and 42 % C (inherited from *B. oleracea*) at this position. In ‘Darmor’, the ‘A’ and ‘C’ variants accounted for 86 and 14 %, respectively. The promotor region harboured higher (5–10-fold) intragenomic heterogeneity than the coding regions (not shown). Using Roche 454 technology we also sequenced 700-bp PCR amplicons comprising the 18S gene (3′ region) and ITS1 regions. Haplotypic analysis of ‘Darmor’ and ‘Yudal’ amplicons revealed shared identical ITS1 major (≥10 % reads) haplotypes. However, these occurred at markedly different frequencies between the two cultivars (Fig. 2C).

### Intragenomic IGS length heterogeneity

**Fig. 3.**
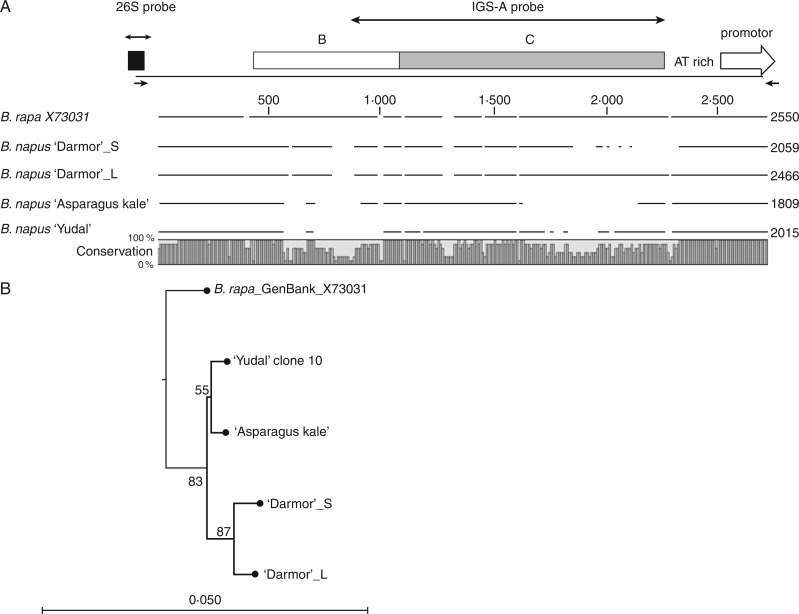
(A) Multiple alignment of IGS sequences from *B. rapa* and *B. napus* A-genome clones. Note multiple indels occur in each clone. The bottom bar indicates conserved and divergent subregions. ‘B’ and ‘C’ represent blocks of subrepeats. The positions of probes and primers are indicated by double and single arrows, respectively. (B) Unrooted neighbour-joining tree reconstructed from IGS sequences. Note both long (‘Darmor’_L) and short (‘Darmor’_S) clones cluster together. Numbers indicate bootstrap support (1000 replicates). The scale bar indicates the number of substitutions per nucleotide positions (5 base change per 100 nucleotide positions).

The highly repetitive nature of IGS makes this region refractory to reconstruct from short Illumina reads. We therefore analysed IGS polymorphisms in several clones from *B. napus* cultivars obtained by cloning of IGS-specific PCR products (see Methods). The organization of aligned A-genome clones is shown in Fig. 3A. The AT-rich region harbouring DNA conformation polymorphisms (Darocha and Bertrand, 1995), core promoter and ∼400 bp downstream of the 26S gene were conserved. In contrast, the long repetitive part was highly variable. In this region multiple indels of variable length were detected, in accordance with variation reported in previous studies (Delseny *et al.*, 1990; Darocha and Bertrand, 1995; Bhatia *et al.*, 1996). In *B. napus* ‘Darmor’, two IGS variants of different lengths were identified: the long (L) 2059-bp variant differed from the short (S) 2066-bp variant in the organization of the C subrepeats. The B subrepeats (according to the nomenclature of Darocha and Bertrand, 1995) and other parts of IGS were invariant. The C subrepeats located proximally to the promoter formed a higher order structure (HOR) of a basic 421-bp unit (Supplementary Data Fig. S2). There were 2·4 copies of HOR units in the long variant of *B. napus* ‘Darmor’ and the *B. rapa* clone (X73031). The short *B. napus* ‘Darmor’ variant and clones from ‘Asparagus kale’ (1809 bp in length) and ‘Yudal’ (2015 bp in length) had only one copy of 421-bp HOR. The divergence between the first and second copy of HOR was 0·73 %, while the third incomplete copy was less conserved (3·4 %). In the phylogenetic tree (Fig. 3B) both short and long variants of *B. napus* ‘Darmor’ clustered on the same branch, indicating a common origin. Thus, individual SNPs rather than IGS length polymorphisms exhibit phylogenetic signals in *Brassica*.

### rDNA homeologue expression in *B. napus* cultivars

**Fig. 4.**
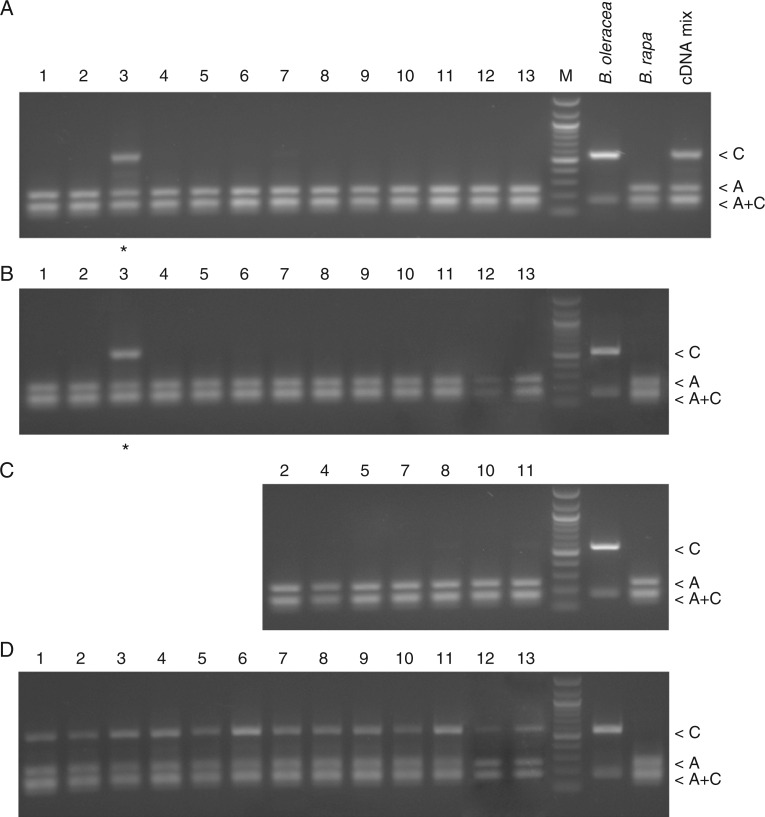
Expression analysis of 35S rDNA. The RT-PCR CAPS was performed using RNA extracted from *B. oleracea* (HDEM) and *B. rapa* (Z1) diploids and natural cultivars of *B. napus*: ‘Darmor’ (1), ‘Taichung’ (2), ‘Norin 9’ (3), ‘Petranova’ (4), ‘Spok’ (5), ‘Asparagus kale’ (6), ‘Norin 6’ (7), ‘Stellar’ (8), ‘Rutabaga’ (9), ‘Loras’ (10), ‘Yudal’ (11), ‘Westar’ (12) and ‘Tapidor’ (13). Gel restriction profiles of ITS1 amplification products obtained from leaf (A), root (B) and flower bud (C) cDNA. Control amplification products from mixed progenitor cDNAs are shown at the right in A. (D) Products from genomic DNA amplification. Asterisks indicate lanes where PCR products corresponding to both homeologues were amplified. The homeologue-specific bands were of ∼250 bp (‘A’, A-genome) and ∼550 bp (‘C’, C-genome) in size. A 100-bp DNA ladder was used as marker (M). The scale bar indicates the number of substitutions per nucleotide positions (5 base change per 100 nucleotide positions).

Expression of homeologous genes was analysed in leaf, root and flower bud tissues by the RT-CAPS method (Fig. 4). In both *B. oleracea* and *B. rapa*, the *Rsa*I enzyme cleaved RT-PCR products into two fragments. The shortest band was shared between both species while the larger 250-bp (marked as C) and 550-bp (A) bands were specific for *B. rapa* and *B. oleracea* progenitors, respectively. In most *B. napus* cultivars, only the A-genome-specific bands were visualized, indicating expression dominance of A-genome NORs. However, in *B. napus* ‘Norin 9’ both A- and C-genome bands were amplified at comparable fluorescence intensity, indicating co-dominance in leaf and root. Control amplification of mixed cDNAs from *B. rapa* and *B. oleracea* showed equal amplification of both homeologues. Thus, the A-genome NORs are always active while those of the C genome are prevalently silenced.

### Genome-specific probes reveal the identity of loci in *B. napus* chromosomes

**Fig. 5.**
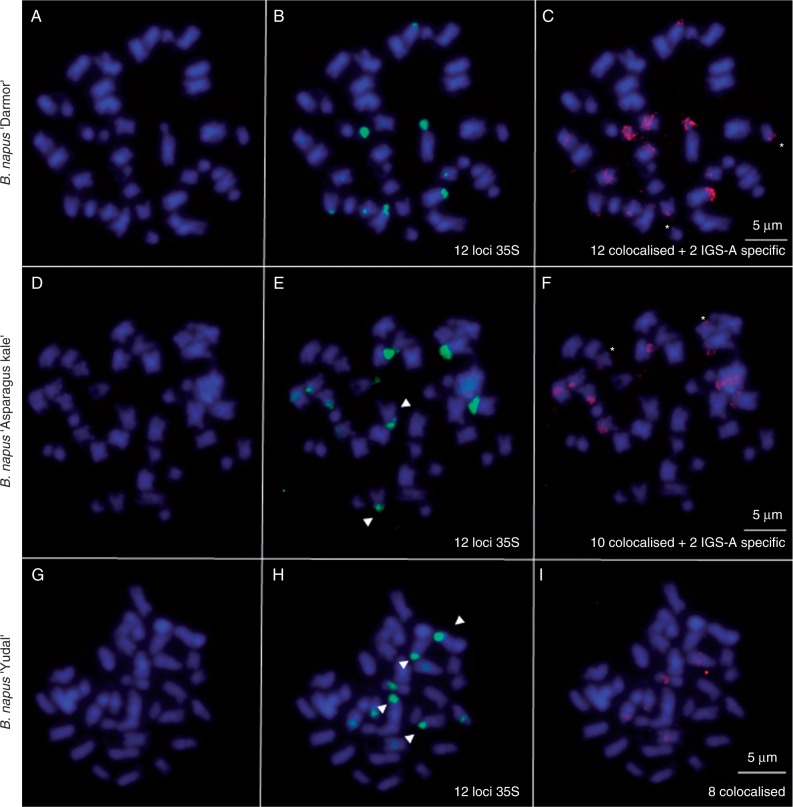
FISH was carried out using 35S rDNA (green) and IGS-A (red) probes. FISH analyses of somatic metaphase chromosomes of *B. napus* ‘Darmor’ (A–C), *B. napus* ‘Aparagus kale’ (D–F) and *B. napus* ‘Yudal’ (G–I). Arrows indicate 35S-specific signals and the IGS-A additional signals are marked by an asterisk. Chromosomes were counterstained with DAPI (blue). Scale bars = 5 μm.

**Fig. 6.**
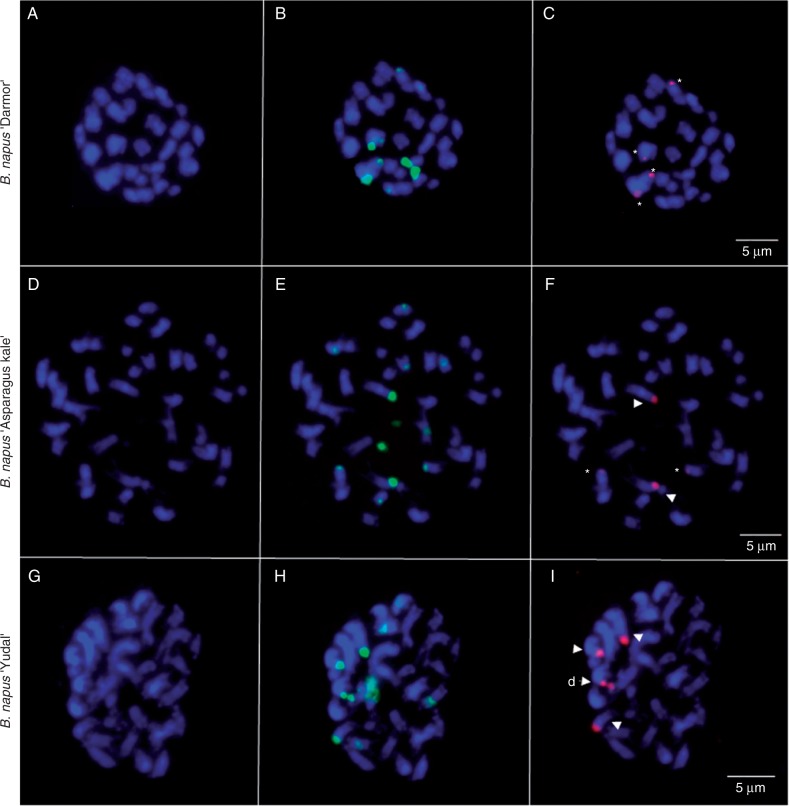
FISH was carried out using 35S rDNA (green) and IGS-C (red) probes. FISH analyses of somatic metaphase chromosomes of *B. napus* ‘Darmor’ (A–C), *B. napus* ‘Aparagus kale’ (D–F) and *B. napus* ‘Yudal’ (G–I). Minor and strong IGS-C signals are marked by asterisks and arrowheads, respectively. A partially decondensed NOR in ‘Yudal’ (I) is indicated by (d-▹). Chromosomes were counterstained with DAPI (blue). Scale bars = 5 μm.

To identify the origin of rDNA loci in *B. napus*, we isolated specific probes for the A-genome and C-genome NORs. The A-genome-specific (IGS-A) and C-genome-specific rDNA probes (IGS-C) were derived from the variable repetitive part of the IGS region having no or little homology with the analogous region in *B. oleracea* or *B. rapa* units, respectively (Fig. 3A). Three *B. napus* cultivars (‘Darmor’, ‘Yudal’ and ‘Asparagus kale’) together with their putative progenitors were analysed by FISH with the 35S rDNA (green), IGS-A (red) and IGS-C (red) probes (Figs 5 and 6).

There were ten and four hybridization sites of the 35S probe in *B. rapa* and *B. oleracea*, respectively (Supplementary Data Figs. S3 and S4). The IGS-A probe hybridized to *B. rapa* chromosomes (six signals) but not *B. oleracea* (Fig. S3). In contrast, the IGS-C probe hybridized to *B. oleracea* but not *B. rapa* chromosomes (Fig. S4).

In *B. napus* ‘Yudal’, the 35S probe hybridized to 12 sites (eight strong and four weak), eight of which co-localized with IGS-A probe (six strong and two weak, Fig. 5I). Four strong 35S sites co-localized with strong IGS-C signals (Fig. 6I).

*Brassica napus* ‘Darmor’ had also 12 35S sites (six strong, four intermediate and two weak). In this cultivar, the IGS-A probe hybridized to 14 sites (Fig. 5C) out of which 12 co-localized with 35S sites; two sites located outside of rDNA. Two strong and two weak 35S sites co-localized with four weak IGS-C signals (Fig. 6C).

In *B. napus* ‘Asparagus kale’, the 35S probe hybridized to 12 sites (six strong and six weak). The IGS-A probe hybridized to 12 sites (Fig. 5F), ten of which co-localized with the 35S signals. The IGS-C probe hybridized to two strong and two weak sites (Fig. 6F), all sites overlapping with the green 35S probe signal.

The *B. napus* cultivars (‘Darmor’, ‘Yudal’ and ‘Asparagus kale’) were also analysed by GISH-like with Bob014O06 (green) and IGS-A (red) probes, which co-localized with the Bob014O06 probe at some sites in ‘ Darmor’ and ‘Asparagus kale’ but not in ‘Yudal’ (Supplementary Data Fig. S5).

## DISCUSSION

There has been large diversification of cultivated *B. napus* round the world since medieval times and especially from the middle of the 20^th^ century (Baranyk and Fábry, 1999). It is therefore interesting to ask how the chromosomes and sequences have evolved under the conditions of intensive breeding. Here, we show that 21 cultivars differ in homeologous rRNA gene ratios, IGS structure and degree of intergenomic homogenization.

### Variable rDNA homeologue ratios among *B. napus* cultivars

In most cultivars of *B. napus*, the A-genome rDNA units were more abundant than the C units. However, three cultivars (‘Norin 9’, ‘Asparagus kale’ and ‘Yudal’) retained a high number of C-genome genes, indicating that the process may still be in progress, as in some recently formed natural allopolyploid populations of *Senecio* (Lowe and Abbott, 1996), *Tragopogon* (Kovarik *et al.*, 2005) and *Cardamine* (Zozomova-Lihova *et al.*, 2014). No apparent relationship between copy number variation and plastid haplotypes was found, indicating that the putative polyphyletic origin plays little or no role in homogenization direction or that breeding schemes crossing the different cultivars did not allow us to identify the origin. Of note, synthetic lines appear to display more cytogenetic and fewer molecular changes than we observed here (Xiong *et al.*, 2011; Ksiazczyk *et al.*, 2011). For example, Xiong *et al.* (2011) observed elimination of major NORs that is not encountered in natural cultivars. It is likely that these highly aberrant genotypes may have arisen in early generations of allopolyploids (Szadkowski *et al.*, 2010; Ksiazczyk *et al*., 2011) while in stabilized species karyotypes with nearly (loss of one of the small loci has been encountered in the A-genome (Hasterok *et al.*, 2006)) additive number of rDNAs seems to be favoured by natural selection.

### Gene conversion of rDNA is a variety-specific phenomenon in *Brassica*

It is striking that several hundred years of intensive cultivation has generated contrasting patterns of rDNA evolution. Both ‘Yudal’ and ‘Darmor’ cultivars of *B. napus* show similar number of major NORs while their genetic composition (units) is vastly different. In *B. napus* ‘Yudal’, we observed a high heterogeneity of units that mostly corresponded to additivity of parental contributions. On chromosomes, intactness of parental loci was evidenced by highly localized hybridization signals of the A-genome- (IGS-A) and C-genome- (IGS-C) specific probes. Thus, in ‘Yudal’, concerted evolution has not been operating at rDNA loci until recently. This pattern contrasts markedly with ‘Darmor’ where concerted evolution apparently homogenized most of the genes to the A-genome type. Based on NGS counts, we estimate that about 65 % of C-genome units (∼900 C-genome copies) were eliminated. On chromosomes, all loci were strongly stained with the IGS-A probe while the IGS-C probe hybridized weakly to four loci, indicating these loci carried both A- and C-genome type units. We do not know whether the different unit types are separated or interspersed within the array. The A/C recombinant ITS sequences were not significantly represented among the NGS reads, suggesting that recombinant genes (if present) did not significantly expand in the genome. The IGS family containing rearranged subrepeats was highly amplified in *B. napus* ‘Darmor’ now comprising most of the rDNA arrays. Also in the coding region about 20 % of highly polymorphic sites appeared to recruit from newly amplified variants. Interestingly, concerted evolution of rDNA in tobacco was associated with rearrangement of IGS (Volkov *et al.*, 1999). Perhaps, interlocus homogenization is preceded by some form of IGS rearrangement and copy number reductions of parental arrays. In *B. napus* ‘Asparagus kale’, the A-genome IGS probe stained all loci except one. In contrast, the C-genome IGS probe hybridized strongly to only one locus, suggesting that the degree of rDNA homogenization is intermediate to ‘Yudal’ and ‘Darmor’ cultivars. This corroborates another study carried out on a different *B. napus* cultivar (Xiong and Pires, 2011) that showed reduced rDNA-FISH signals on the C-genome (compared to the *B. oleracea* progenitor), supporting the hypothesis that the gene richness of C-genome NORs tends to decrease in most natural *B. napus*. Partial replacement of C-genome genes by A-genome genes potentially explains why *B. oleracea* IGS was unable to block completely GISH signals on C- genome NORs (Howell *et al.*, 2008).

### Dominance of A-genome nucleolar expression

In natural *B. napus*, epigenetic variability in rRNA expression was much less pronounced than the variability in copy numbers. The majority (95 %) of cultivars showed strong silencing of C-genome genes (A-genome nucleolar dominance) consistent with a previous report (Chen and Pikaard, 1997). The direction of epigenetic silencing in natural *B. napus* is similar to that observed in synthetic *B. napus* (Ksiazczyk *et al.*, 2011), indicating that silencing established early in evolution is relatively stable. None of the cultivars showed reverse dominance, i.e. silencing of A-genome NORs. Activation of silent C-genome genes leading to co-dominant phenotype has been reported in certain organs, such as adventious roots (Hasterok and Maluszynska, 2000) or flower organs (Chen and Pikaard, 1997). In our experiments, flowers did not show markedly elevated expression of the C-genome genes and only very faint bands were detected in a minority (15 %) of flower samples after RT-CAPS (data not shown). Nevertheless, we cannot exclude the possibility that certain cell types, not analysed in this study, may specifically express C-genome genes. Partial decondensation of one of the C-genome loci in a ‘strongly’ A-genome-dominant *B. napus* ‘Yudal’ (Fig. 6I) is consistent with this hypothesis. The co-dominant phenotype in ‘Norin 9’ is intriguing as it occurs irrespective of organ specificity. Perhaps, silencing established at early generations may occasionally be reversed during population divergence.

### Why are the A-genome units so invasive?

We observed replacement of C-genome loci by the A-genome units while we did not observe the reverse situation where the C-genome units would be found on the A-genome chromosomes. The question arises as to why the A-genome units are so penetrant and prone to colonize partner chromosomes. There are several hypotheses:

(1) rDNA may follow an overall genomic trend of gene conversion events that appear to be more frequent from the A to C genome than from the C to A genome (Chalhoub *et al.*, 2014). The mechanism of gene conversion remains obscure, but it is believed to involve some form of meiotic recombination (Eickbush and Eickbush, 2007). Indeed, homeologous recombination has been considered as a major source of genetic variability in *B. napus* (Nicolas *et al.*, 2007; Gaeta and Pires, 2010; Szadkowski *et al.*, 2010, 2011). In support of this, four cultivars (‘Darmor’, ‘Nachan’, ‘Maxol’ and ‘Taichung’) harbouring predominantly the A-genome genes (Fig. 1D) belong to a group showing frequent homeologous recombination (Cifuentes *et al.*, 2010). However, major A- and C-genome NORs occur on non-homeologous chromosomes that pair rarely in meiosis and interlocus recombination is far less frequent than intralocus recombination in *Drosophila* (Schlotterer and Tautz, 1994). Nevertheless, some form of non-homeologous recombination cannot be excluded due to segmented homology detected between different chromosomes (Parkin *et al.*, 2014). Interlocus homogenization of NORs at non-homeologous chromosomes was also reported in other systems (Kovarik *et al.*, 2004), suggesting the existence of other recombination mechanisms than those acting during meiosis. In *Arabidopsis*, ectopical rDNA loci physically associate in interphase at a frequency that is higher than random (Pecinka *et al.*, 2004). Hence a translocation of arrays or its part to another position may occur in interphase, perhaps during nucleolar fusions (Kovarik *et al.*, 2008).

(2) Structural features of IGS should be considered. The IGS regions of many plants evolve astonishingly quickly (Saghai-Maroof *et al.*, 1984; Borisjuk *et al.*, 1997; Carvalho *et al.*, 2011; Coutinho *et al.*, 2016). For example, in *B*. *napus* progenitor species that diverged less than 3 Mya (Inaba and Nishio, 2002), the rDNAs not only changed the position on chromosomes but also evolved species-specific IGS variants (Bhatia *et al.*, 1996; Delseny *et al.*, 1990; Bennett and Smith 1991). The long (>1·8 kb) region of the A-genome IGS is composed of 21- and 28-bp GC-rich subrepeats which are among the shortest in plants and which form higher order structures (Fig. 3 and Supplementary Data Fig. S2). Given that gene conversion is biased towards GC-rich sequences (Escobar *et al.*, 2011) it may be that novel IGS variants are generated frequently. In accordance with this idea, we identified the A-genome IGS subrepeats as rDNA-independent loci in two *B. napus* cultivars (‘Darmor’ and ‘Asparagus kale’), undergoing intergenomic homogenization of rDNAs. The solitary IGS loci have been regularly detected in different systems (Stupar *et al.*, 2002; Lim *et al.*, 2004) forming independent satellites of unknown function. We propose that these non-rDNA IGS sites could be a hallmark of recombination events within the NORs possibly arising by integration of extrachromosomal covalent circles (Navratilova *et al.*, 2008).

(3) Epigenetic modification directs homogenization. We previously argued that silent epigenetically modified units may be less vulnerable to homogenization than active units (Kovarik *et al.*, 2008). Heterochromatic marks including histone H3K9 dimethylation and increased cytosine methylation acquired at early generations of synthetic wheat lines lead to their elimination from the tgenome in advanced generations (Guo and Han, 2014). *Brassica napus* diploid progenitors also differ in number and composition of repeats (Cheung *et al.*, 2009) and epigenetic landscape of their genomes (Braszewska-Zalewska *et al.*, 2010). In synthetic lines of *B. napus*, we observed immediate silencing of C-genome NORs accompanied by enhanced methylation of promoter regions (Ksiazczyk *et al.*, 2011). Perhaps, long-term silencing of most C-genome units led to their gradual elimination from the genome. In contrast, an open chromatin configuration of intensively transcribed A-genome units may predispose them to homogenization across the genome.

## CONCLUSIONS

Patterns of rDNA sequence conversion and provenance of the lost loci are highly idiosyncratic and differ even between allopolyploids of identical parentage, indicating that allopolyploids deriving from the same lower-ploidy-level parental species can follow different evolutionary trajectories (Wendel *et al.*, 1995; Weiss-Schneeweiss *et al.*, 2012). Here, we identified this trend even in relatively recently formed *B. napus*. We observed shifts in gene ratios and identified intergenomic gene conversion events that partially replaced genes in partner loci. The resulting rDNA homogenization seems to act in a cultivar-specific manner with some preference towards one parental subgenome. This bias is possibly influenced by epigenetic status established early in the allopolyploid nucleus formation. Stabilization of the allopolyploid nucleus is an ongoing process that is accompanied by evolution of distinct rDNA genotypes.

## SUPPLEMENTARY DATA

Supplementary data are available online at www.aob.oxfordjournals.org and consist of the following. Table S1: list of Brassica accessions and their analysis. Figure S1: southern blot hybridization of genomic DNA from several *B. napus* cultivars. Figure S2: structural features of IGS in ‘Darmor’ long and short units. Figure S3: validation of the A-genome probe specificity by Southern hybridization and FISH. Figure S4: validation of the C-genome probe specificity by Southern hybridization (A) and FISH (B). Figure S5: dispersion of A-genome units across the chromosomes analysed by FISH.

Supplementary Data
