# Supplementary material for: Gene conversion events and variable degree of homogenization of rDNA loci in cultivars of Brassica napus
Source: Ann Bot. 2016 Oct 5;119(1):13–26. doi: 10.1093/aob/mcw187 (PMC5218374; doi:10.1093/aob/mcw187)
Supplement: Supplementary Data [file supp_119_1_13__index.html]

Gene conversion events and variable degree of homogenization of rDNA loci in cultivars of Brassica napus — Supplementary Data 

# Gene conversion events and variable degree of homogenization of rDNA loci in cultivars of *Brassica napus*

## Supplementary Data

files

- Supplementary Data - zip file
