# Supplementary material for: Gene conversion events and variable degree of homogenization of rDNA loci in cultivars of Brassica napus
Source: Ann Bot. 2016 Oct 5;119(1):13–26. doi: 10.1093/aob/mcw187 (PMC5218374; doi:10.1093/aob/mcw187)
Supplement: Supplementary Data [file supp_mcw187_suppl_data.zip › aob-16376-s07.docx]

**Figure S1**. Southern blot hybridization of genomic DNA from several *B. napus* cultivars. In the right (**B**) panel the blot was hybridized with the C-genome IGS probe. After stripping the blot was rehybridized with the 26S probe (**A**). ´C´-C-genome bands; ´A´-A-genome bands; ´A*´ indicates an IGS family amplified in a subset of *B. napus* cultivars. Note, differences in hybridization profiles between ‘Norin 9’ and other ‘Norin’ cultivars. Note, strong hybridization of the C-genome IGS probe to ‘Yudal’ and ‘Norin 9’ DNAs.

**Figure S2**. Structural features of IGS in ‘Darmor’ long and short units. The long family expanded the C subregion composed of short GC-rich (65 %) subrepeats. The higher order 421-bp repeat (HOR) is shown below the conservation plot. Each unit is composed of several tandemly arranged 21- (brown) and 28- (green) subrepeats plus their truncated versions. The rest of the sequence was >95% identical.

**Figure S3**. Validation of the A-genome probe specificity by Southern hybridization (A) and FISH (B). In (**A**) the 26S probe hybridized to both *B. oleracea* and *B.* *rapa* fragments. The IGS-A probe hybridized to *B. rapa* fragments only. (**B**) FISH to *B. oleracea* (a-b) and *B. rapa* (c-d). Note, absence of A-genome IGS signals on *B. oleracea* chromosomes. Chromosomes were hybridized *in situ* with the IGS-A probe (red) and 45S rDNA (green). Probes were washed at 42°C in 50% formamide 0.2 X SSC (98% stringency). Chromosomes were counterstained with DAPI (blue). Bar represents 5 µm.

**Figure S5**. Dispersion of A-genome units across the chromosomes analysed by FISH. Somatic metaphase chromosomes of *B.* *napus* ‘Darmor’ (**A**), ´Asparagus kale´ (**B**) and ´Yudal´ (**C**) were hybridized with the IGS-A (in red) and Bob014O06 probes (in green). The Bob014O06 showed a GISH-like signal on all C-genome chromosomes. The A-genome IGS signals on the C-genome chromosomes are indicated by (+) and the additional signals by (*). Chromosomes carrying the intact C-genome NORs are indicated by arrowheads. Bar represents 5 µm.
