# Supplementary material for: Gene conversion events and variable degree of homogenization of rDNA loci in cultivars of Brassica napus
Source: Ann Bot. 2016 Oct 5;119(1):13–26. doi: 10.1093/aob/mcw187 (PMC5218374; doi:10.1093/aob/mcw187)
Supplement: Supplementary Data [file supp_mcw187_suppl_data.zip › aob-16376-s02.docx]

|  | Accession | Southern hybridisation | FISH | RT-PCR, (root, leaf and flower) | Genomic SNP analysis | IGS cloning |
| --- | --- | --- | --- | --- | --- | --- |
| *B. napus* | Asparagus kale | + | + | +,+,- | - | + |
|  | Brutor | + | - | - | - | - |
|  | Darmor | + | + | +,+,- | + | + |
|  | Loras | + | - | +,+,- |  | - |
|  | Maxol | + | - | - | - | - |
|  | Mohican | + | - | - | - | - |
|  | Nachan | + | - | - | - | - |
|  | Norin 1 | + | - | - | - | - |
|  | Norin 6 | + | - | +,+,+ | - | - |
|  | Norin 9 | + | - | +,+,- | - | - |
|  | Norin 10 | + | - | - | - | - |
|  | Oro | + | - | - | - | - |
|  | Petranova | + | - | +,+,+ | - | - |
|  | Rutabaga 22 | + | - | - | - | - |
|  | Rutabaga 95 | + | - | +,+,- | - | - |
|  | Spok | + | - | +,+,+ | - | - |
|  | Stellar | + | - | +,+,+ | - | - |
|  | Taichung | + | - | +,+,+ | - | - |
|  | Yudal | + | + | +,+,+ | + | + |
|  | Tapidor | + | - | +,+,- | - | - |
|  | Westar | + | - | +,+,- | - | - |
| *B. rapa* | Z1 | + | + | +,+,+ | - | - |
|  | NRC Saskatoon, Canada | - | - | - | + | - |
|  | subsp. pekinensis | - | - | - | + | - |
| *B. oleracea* | HDEM | + | + | +,+,+ | - | - |
|  | NRC Saskatoon, Canada | - | - | - | + | - |

Table S1. *List of Brassica accessions and their analysis*.
