# Supplementary figures and images for: Gene conversion events and variable degree of homogenization of rDNA loci in cultivars of Brassica napus
Source: Ann Bot. 2016 Oct 5;119(1):13–26. doi: 10.1093/aob/mcw187 (PMC5218374; doi:10.1093/aob/mcw187)

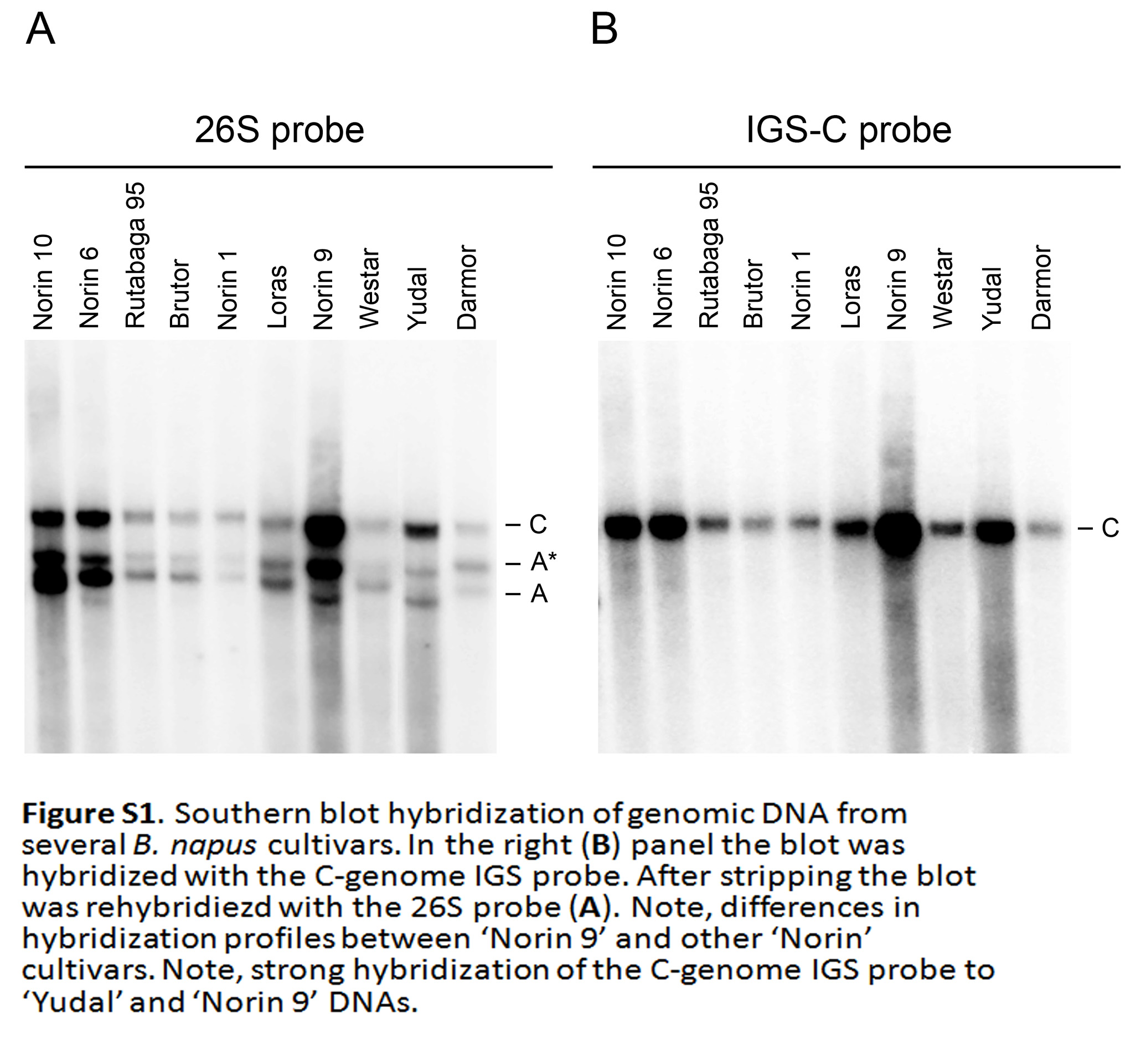

Supplement: Supplementary Data [file supp_mcw187_suppl_data.zip › aob-16376-s03.jpg]

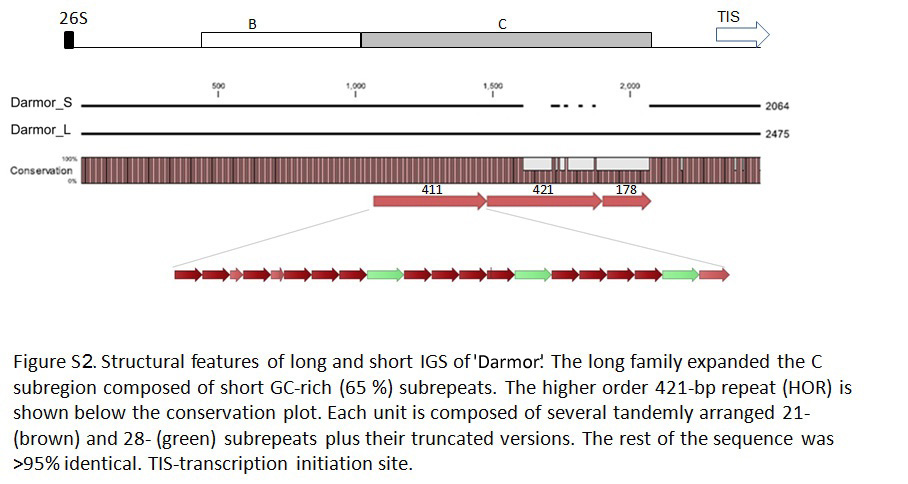

Supplement: Supplementary Data [file supp_mcw187_suppl_data.zip › aob-16376-s04.jpg]

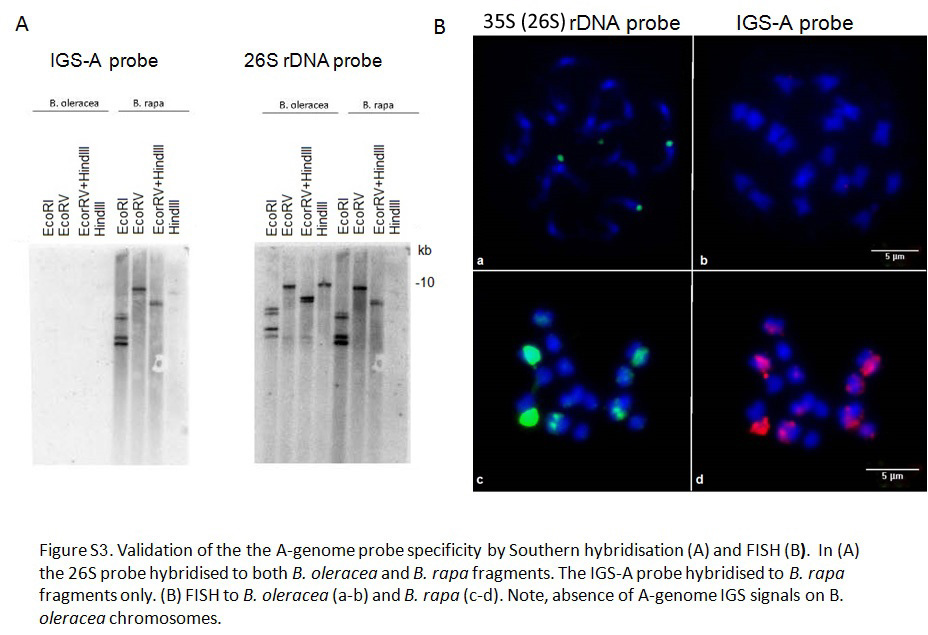

Supplement: Supplementary Data [file supp_mcw187_suppl_data.zip › aob-16376-s05.jpg]

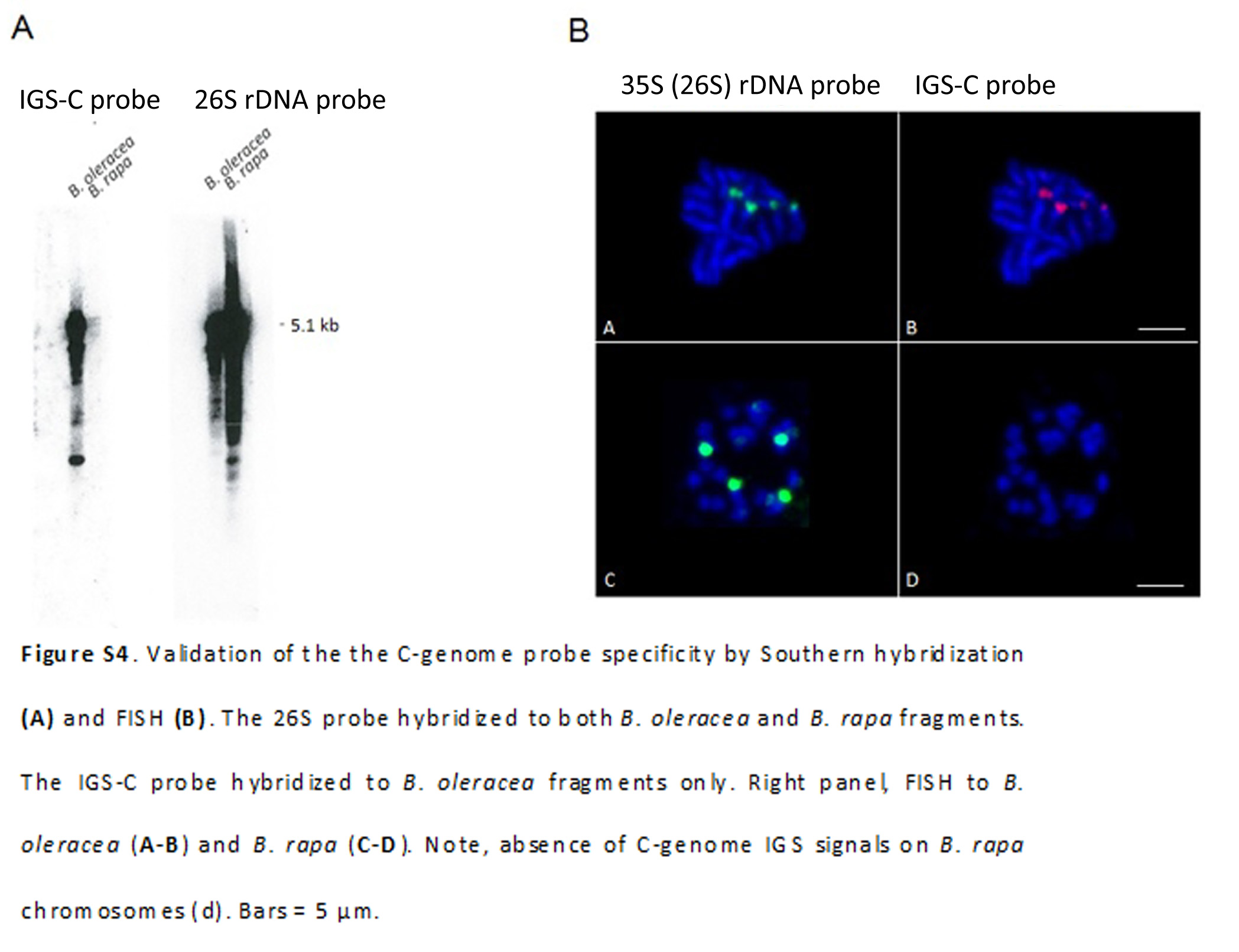

Supplement: Supplementary Data [file supp_mcw187_suppl_data.zip › aob-16376-s06.jpg]

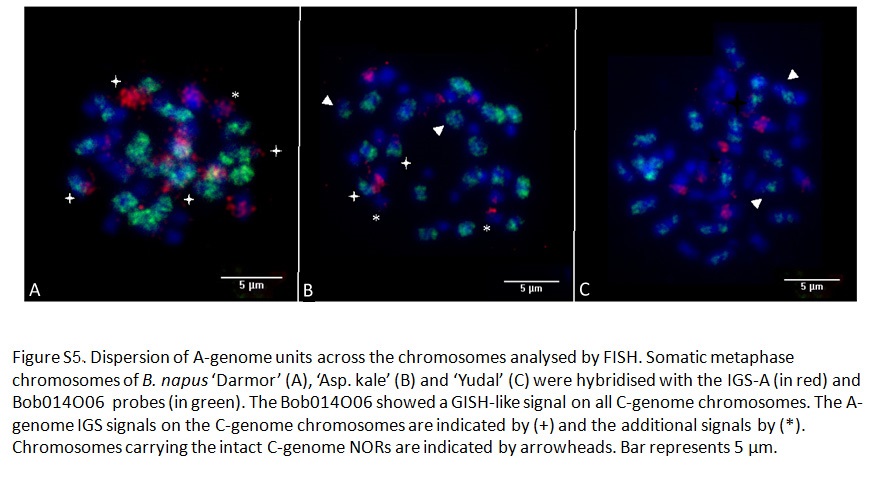

Supplement: Supplementary Data [file supp_mcw187_suppl_data.zip › aob-16376-s01.jpg]
